# Shoulder injuries in professional rugby: a retrospective analysis

**DOI:** 10.1186/1749-799X-8-9

**Published:** 2013-04-26

**Authors:** Ian G Horsley, Elizabeth M Fowler, Christer G Rolf

**Affiliations:** 1 Centre for Rehabilitation and Human Performance Research, University of Salford, Fredrick Road, Salford M6 6PU, UK; 2 Department of Orthopaedic Surgery, Clintec, Karolinska University Hospital, Karolinska Institutet, Stockholm 171 77, Sweden; 3 English Institute of Sport, Sports City, Gate 13, Rowsley Street, Manchester M11 3FF, UK

**Keywords:** Rugby, Shoulder, Superior labrum, Arthroscopy

## Abstract

**Background:**

In the literature, little is known about the level and pattern of rugby injuries. Of the shoulder injuries reported, 51% of these are caused during a tackle, and 65% of all match injuries affected the shoulder.

**Objective:**

The study aims to describe a sport-specific unique intra-articular shoulder pathology of professional rugby players, who presented with persistent pain and dysfunction despite physiotherapeutic treatment and rest.

**Method:**

This study is a retrospective analysis set at a university sports medicine clinic. Eighty-seven professional rugby players, referred by their professional medical team since they could no longer play, underwent shoulder arthroscopy between June 2001 and October 2007 due to persistent shoulder pain and dysfunction. All were full-time professional male rugby union and rugby league players. They all had failed conservative treatment for their complaint, and the diagnosis was unclear. Arthroscopic findings were used as a measure of main outcome.

**Results:**

The primary mechanism of injury was reported as direct tackling (56%; *n* = 49) followed in succession by falling onto the arm (10%; *n* = 8). However, in 30% of the cases, no definite injury could be recalled. The main operative finding was that most patients exhibited multiple shoulder pathologies, with 75% of cases presenting with two or more pathologies. A superior labrum anterior to posterior (SLAP) lesion was evident at arthroscopy in 72 of the 87 cases (83%), while rotator cuff tears were evident in 43% of cases (*n* = 37). One-third of all cases had a Bankart tear (*n* = 29), despite none of them reporting previous dislocations, while other labral tears, excluding SLAP tears, to the inferior or posterior labrum were present in 34% (*n* = 30) of the cohort.

**Conclusions:**

Repeated tackling, which is clearly rugby specific, is most likely to be responsible for most of these shoulder injuries, which upon arthroscopic examination, showed signs of mixed pathology. We suggest that an early arthroscopic investigation is valuable in this population in order to confirm treatable diagnosis on the painful shoulder and expedite a safe return to play.

## Introduction

There has been an increase in the frequency and severity of shoulder injuries among rugby players in recent years [1]. This may be because the game has become more aggressive and intense, and over the years, the game has changed from being largely an amateur sport to one that is played at a professional level [1]. The mean incidence of injuries recorded from three studies within a professional rugby union is 86.4 injuries per 1,000 player hours [2]. Pooled data analysis of injury incidence in a rugby league found an overall injury rate of 40.3 injuries per 1,000 player hours [3]. During one season studied, the incidence of shoulder injuries was significantly lower during training (0.10/1,000, player/training hours) compared with matches (8.9/1,000 player/match hours), and the number of days lost to training or playing due to reported shoulder dislocation or instability was 176 days per 1,000 player hours [4]. This sport is unique in its rules and ways of tackling, and one could expect that a typical pattern of injuries would follow the repeated tackling in training and games. A typical personal trait for professional rugby players is also a generally high pain threshold. Players often continue to play despite niggles and minor injuries. Consequently, when players seek medical advice and complain to the extent that they cannot play due to shoulder pain and dysfunction [5], from experience, one would expect a pathoanatomic correlate. But what is the pathoanatomical consequence for a shoulder joint which is tackled and tackles day out and in over years? Similarly, it is well recognized that the application of muscular rugby shoulders provides a challenge for even the most experienced clinician. Over the years, our team has seen and treated a large number of such players. In cases where arthroscopic surgery is indicated in this cohort, it is our experience that the clinical history and examination often do not fully reflect the pathology visualized from the scope. The purpose of this study was to highlight an observed ‘typical’ pattern of injuries to be observed when treating a professional rugby player with shoulder pain and dysfunction.

## Methods

The subjects of this study were 87 professional rugby players who underwent shoulder arthroscopy due to ongoing pain and dysfunction between June 2001 and October 2007. All were full-time professional male rugby union and rugby league players. They were all referred by a member of the medical team at their respective clubs, having failed conservative treatment for their complaint. Upon initial presentation at the clinic, a senior orthopedic surgeon conducted a systematic physical examination of the patient, recording the player’s age; type, side, severity, and mechanism of injury; and time since onset of symptoms. Following this, a standardized battery of clinical tests was routinely carried out, during which any patient symptoms elicited by the test were recorded. The physical examination typically consisted of assessing the bilateral active and passive range of motion of the shoulders to determine pain, range of motion, and end feel. Finally, specialized clinical tests specific to the shoulder were carried out, and these were as follows: Hawkin’s impingement test, internal impingement test, O’Brien’s test, Gerber’s lift-off test, across body test, apprehension-relocation test, palm-up test, Jobe’s test, and sulcus sign (Table 1). These specific tests were utilized as they were routine tests carried out within the clinic which had previously been evaluated for diagnostic accuracy within a cohort of recreationally active athletes [15]. All players underwent X-rays, and many of them (although not detailed here) underwent MRI investigation. Following clinical examination, a clinical working diagnosis was made, and where deemed appropriate, radiological investigations were undertaken. In all these cases, shoulder arthroscopy was deemed indicated for further evaluation and treatment.

**Table 1:** Clinical tests of the shoulder for the clinical examination

**Test**	**Description**	** Reference**
O’Brien’s test	The arm is forward-flexed to 90° with the elbow in full extension and is then adducted 10° to 15° medial to the sagittal plane of the body. The forearm is then pronated, and the arm is internally rotated so that the thumb points downward. The physician applies a downward force to the arm and, while maintaining the overall position of the arm, supinates the arm and repeats the maneuver.	O’Brien et al. [6]
	The test is positive if the patient experiences pain during the first maneuver and the pain decreases or disappears with the second.	
Jobe’s test	The patient places both arms in 90° abduction and 30° horizontal adduction, in the plane of the scapula, with his thumbs pointing downward in order to produce medial rotation of the shoulder; the examiner then pushes the patient’s arms downward while asking the patient to resist the pressure. Inability to resist despite pain denotes tendonitis.	Jobe and Jobe [7]
Hawkins-Kennedy test	The patient raises the arm forward to 90°, while the examiner forcibly internally rotates the shoulder. Pain with this maneuver suggests subacromial impingement or rotator cuff tendonitis.	Hawkins and Kennedy [8]
Palm-up test	The patient is asked to elevate the arm anteriorly against resistance, with the elbow extended and the palm facing upward. The test is positive if the patient feels pain at the anterior aspect of the arm along the course of the long head of the biceps brachii.	Gilcreest [9]
Compression-rotation test	The shoulder is placed at 45° of abduction. The clinician stabilizes the superior portion of the shoulder with one hand and grasps the elbow in the other. The distal hand applies a compressive force up the long axis of the humerus toward the superior labrum. While compressing the humerus cranially, a concurrently produced clockwise and counterclockwise circumduction is performed in an attempt to entrap a piece of labrum between the humeral head and the glenoid fossa. The patient’s complaint of pain, snapping, or catching sensations is considered a positive test for a superior labral tear or ‘superior labrum anterior to posterior’ (SLAP).	Snyder et al. [10]
Apprehension-relocation test	With the patient lying supine on the examination table, the clinician stands along the patient’s affected side and abducts the patient’s arm to 90°, flexes the elbow to 90°, and externally rotates the shoulder slowly. A positive test is indicated by a look or feeling of apprehension or alarm on the patient’s face and the patient’s resistance to further motion at the glenohumeral joint; application of a posteriorly directed force to the humeral head will remove the patient’s anxiety.	Rowe and Zarins [11]
Across body test	The arm is brought to 90° of forward flexion and then passively brought across the front of the body. The test is positive if pain is elicited at the anterior shoulder, indicating a possible subcoracoid bursitis or labral/capsular tear.	Sillman and Hawkins [12]
Gerber’s lift-off test	The patient is asked to place one hand against the back at the level of the waist with the elbow in 90° flexion. The examiner pulls the hand to about 5 to 10 cm from the back while maintaining the 90° bend in the elbow.	Gerber and Krushell [13]
	The patient is then asked to hold the position without the examiner’s help.	
	This test is positive if the hand cannot be lifted off the back, detecting complete rupture of the subscapularis tendon.	
Sulcus sign	With the patient’s arm positioned at 0° of abduction, the clinician grasps the patient’s relaxed arm just distal to the elbow on the dorsal surface of the forearm and applies a gentle, inferiorly directed force, parallel to the long axis of the humerus. In patients with increased glenohumeral laxity, a sulcus sign will appear just inferior to the acromion.	Neer and Foster [14]

All data were prospectively documented in a standardized database, and arthroscopic procedures were systematically recorded on DVD. Clinical examination and arthroscopy were all performed by the same consultant orthopedic surgeon.

## Ethical considerations

Consent to use their clinical data was obtained in writing from each patient, and ethical approval for the study was obtained from the Ethical Committee at the University of Sheffield, UK.

## Results

The mean age at the time of initial consultation was 25.5 years (±4.5 years, range 17 to 36 years). The age range that dominated the sample population of this study was between 26 and 30 years of age, as 30% of the cohort were within this age bracket. The age range least represented was that above 30 years of age.

The majority of patients originated from a rugby union (67%), with the remaining being rugby league players. Just over half the injuries were of sudden, acute onset (*n* = 44) with 31% of these presenting within 4 weeks from injury onset. The primary mechanism of injury reported was direct tackling (56%, *n* = 49) followed in succession by falling onto the arm (10%, *n* = 8). A large portion of players, however, (30%, *n* = 26) were unable to recall a specific event which caused the injury. Activity-related and movement-related pain was reported in all cases, with nine cases citing instability as a clinical complaint in conjunction with pain. A feeling of weakness in certain positions of the shoulder was reported in 45% of cases. The average time from injury to initial consultation for the entire group was 19.7 weeks (±39 weeks), with all players receiving physiotherapy from their respective team medical staff prior to this. All arthroscopies were subsequently conducted within an average of 3.6 weeks (SD 6.7 weeks) following initial consultation.

Surgery was performed on the right shoulder in 48 cases, and normal arthroscopy was reported in one case. No complications were reported in any of the arthroscopies undertaken. The main operative finding (Table 2) was that most patients exhibited multiple shoulder pathologies, with 75% of cases presenting with two or more pathologies. A SLAP lesion was evident at arthroscopy in 72 of the 87 cases (83%), while partial rotator cuff tears were evident in 43% of cases (*n* = 37). One-third of all cases had a Bankart tear (*n* = 29), while other labral tears, excluding SLAP tears, to the inferior or posterior labrum were present in 34% (*n* = 30) of the cohort. The presence of a SLAP lesion with concomitant rotator cuff damage was the most common multiple pathology evident at surgery, present in 16% of all cases. Of the total 87 arthroscopies conducted, a quarter of these cases (25%) had an isolated pathology, with SLAP lesion being the most common isolated pathology (64% of all isolated pathologies only). Sole injury to either the rotator cuff, labrum, or biceps was rare, although this may be a function of the mechanism of injury.

**Table 2:** Arthroscopic findings in 87 consecutive shoulder surgeries in professional male rugby players in the UK

**Operative findings**	**Number of cases**
Normal arthroscopy	1
Isolated SLAP tear	14
Isolated Bankart injury	3
Isolated partial rotator cuff tear	2
Isolated labral damage (non-SLAP)	2
Isolated partial biceps tear	1
Bankart lesion with other labral tear	9
Bankart lesion and partial rotator cuff tear	1
Bankart lesion and Hill-Sachs lesion	2
SLAP tear and partial rotator cuff tear	14
SLAP tear and other labral damage	8
Rotator cuff tear and other labral damage (non-SLAP)	2
Mixed pathology (three or more identified pathologies)	28

**Figure 1:**
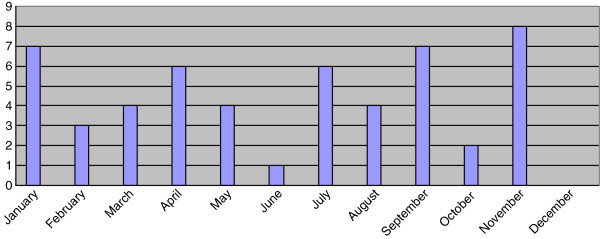
Presentation of injuries over the season.

**Figure 2:**
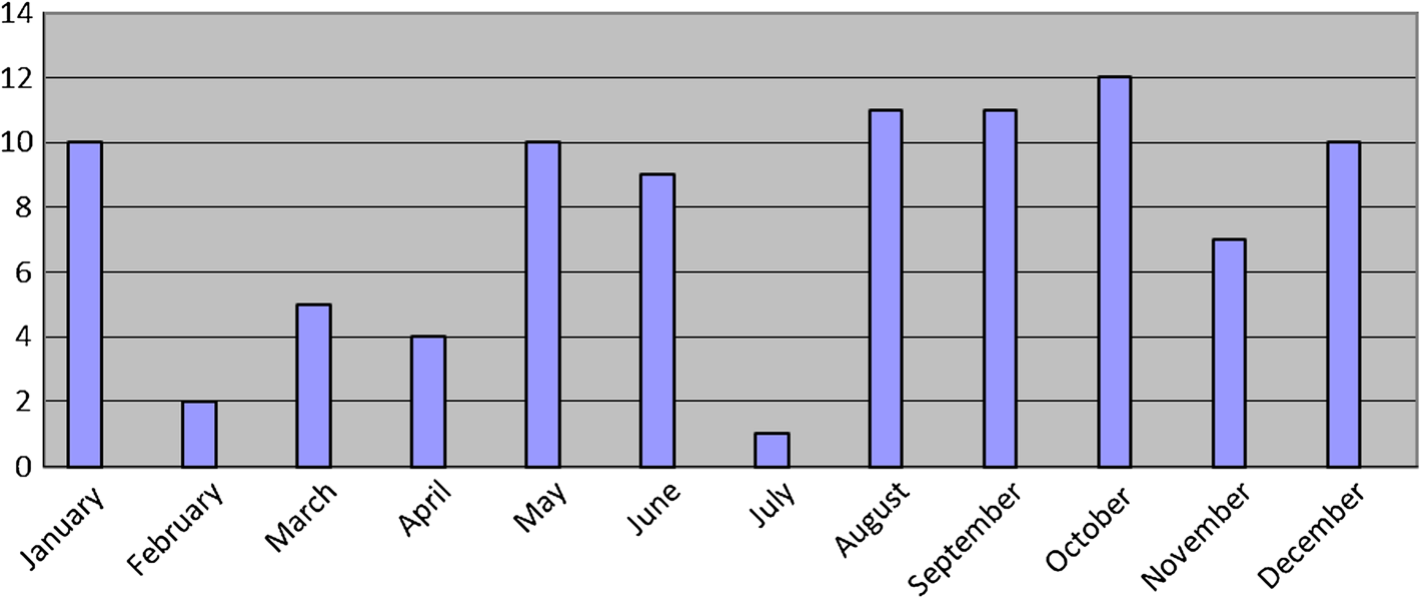
Surgical procedures over the season.

The time of year when professional rugby union and rugby league players present for medical attention for shoulder injuries is broadly spread equally across the year for both rugby codes (Figures 1 and 2). Within the rugby league, the majority of injuries occurred in May (*n* = 4), closely followed by November (*n* = 3) and December (*n* = 2); the latter months being times in the year which correspond to pre-season ‘friendly’ games being undertaken. The month of May is approximately halfway through the competitive season rugby league. Within the rugby union, January, being approximately the midpoint of the season, was the month when the majority of players reported injury to their shoulder occurring (*n* = 6). May and September followed in close succession (*n* = 5 and *n* = 4, respectively), two months representing the beginning and end of the rugby union season in the UK.

## Discussion

This study identifies the multifactorial nature of intra-articular pathology reflecting the morbidity from shoulder injuries in professional rugby players. The study reported that none of these players had an isolated injury, but the pathology observed indicated a sequential number of injuries adding onto each other over time. Whether this is due to one initial insufficiently rehabilitated injury and secondary injuries or non-coincidental injuries from different unrelated tackling events is not known. It is also notable that so many players presented with Bankart injuries despite having no recollection of frank dislocation of their shoulder. Earlier studies in professional rugby have identified a high incidence of shoulder injuries in professional rugby [15-18] (Le Roux, unpublished data) and have also identified tackling as being responsible for a majority of reported shoulder injuries, also in accordance with previous studies [18-28] (Le Roux, unpublished data). Funk and Snow [29] in their retrospective study of 18 professional rugby players identified tackling as being responsible for an injury in 85% of players.

Within this study, arthroscopy appears to demonstrate a sport-specific injury pattern. Funk and Snow [29] described two specific arm positions at contact, either abducted and externally rotated or adducted and associated specific injuries to each position. This study did not make those distinctions due to the fact that 30% of subjects could not recall a definitive mechanism of injury.

Seventy-five percent of the subjects presented with two or more pathologies within the shoulder, which could be accounted for by the relatively long time from onset of symptoms to presentation at the sports medicine clinic (19.7 ± 39 weeks). This could possibly account for the multiple pathologies which were found on arthroscopy: an initial injury causing loss of passive stabilization to the glenohumeral joint, which then produced micro-instability and repeated accumulated trauma within the joint, which reflects the findings of previous research in the other overhead sports that failure to diagnose leads to progression of injury [30]. The presence of any of the injuries reported in isolation could be responsible for the instability at the glenohumeral joint and could explain the fact that activity-related pain was the most consistently reported symptom.

Pain causes active inhibition of slow motor unit recruitment within the local stabilizing muscles around the glenohumeral joint. According to Sahrmann, when a global imbalance between the single joint stabilizer muscle and the two joint mobilizing muscles occurs at the glenohumeral joint, there is a loss of neuromuscular control which results in poor control of the humeral head centering on the glenoid [31]. The disruption of the passive stability system of the glenohumeral joint (capsule and labrum) and an associated local muscle dysfunction would be responsible for the dynamic instability which was reported as activity-related pain, and could also explain why there was an apparent low success rate with conventional physiotherapy. Implications of a local system dysfunction are characterized by a high incidence of recurrence and pain [32,33].

Successful return to unrestricted function requires integrating the appropriate diagnosis, surgical management, and rehabilitation [34]. We would suggest that arthroscopy should be considered early in professional rugby players who present with activity-related pain of a magnitude that stops them from playing, especially those players who identify tackling as being responsible for the cause of their shoulder pain. The arthroscopic treatment does not differ from that of other patients with similar injuries and is not considered in detail in this article.

## Conclusion

Shoulder injuries in professional rugby players are responsible for a high morbidity. Tackling has been shown to be responsible for a large number of these injuries, with players presenting with symptoms of activity-related pain and, upon arthroscopic examination, showing signs of a mixed pathology. We suggest that early arthroscopic investigation be carried out in this population in order to understand the injury pattern, expedite their treatment and return to play, and reduce the possibility of further damage to the shoulder joint.

## Competing interests

The authors declare that they have no competing interests.

## Authors’ contributions

IGH reviewed the database and prepared the manuscript. EMF carried out the statistical analysis and assisted with preparation of the manuscript. CGR assembled the database, carried out the surgeries, and supervised the writing of the whole paper. All authors read and approved the final manuscript.
